# Network pharmacology-based strategy for predicting therapy targets of *Tripterygium wilfordii* on acute myeloid leukemia

**DOI:** 10.1097/MD.0000000000023546

**Published:** 2020-12-11

**Authors:** Tingting Fang, Lanqin Liu, Wenjun Liu

**Affiliations:** a Department of Pediatric Hematology, The Affiliated Hospital of Southwest Medical University; b Birth Defects Clinical Research Center of Sichuan Province, Luzhou, Sichuan, China.

**Keywords:** active compounds, anti-AML, network pharmacology, *Tripterygium wilfordii*

## Abstract

This is a study on the potential therapeutic targets and pharmacological mechanism of *Tripterygium wilfordii* (TW) in acute myeloid leukemia (AML) based on network pharmacology.

Active components of TW were obtained by network pharmacology through oral bioavailability, drug-likeness filtration. Comparative analysis was used to investigate the overlapping genes between active ingredient's targets and AML treatment-related targets. Using STRING database to analyze interactions among overlapping genes. Both KEGG pathway analysis and Gene Ontology enrichment analysis were conducted in DAVID. These genes were analyzed for survival in OncoLnc database.

We screened 53 active ingredients; the results of comparative analysis showed that 8 active ingredients had an effect on AML treatment. On the basis of the active ingredients and overlapping genes, we constructed the Drug-Compounds-Genes-Disease Network. Survival analysis of overlapping genes indicated that some targets possessed a significant influence on patients’ survival and prognosis. The enrichment analysis showed that the main pathways of targets were Toll-like receptor signaling pathway, NF-kappa B signaling pathway, and HIF-1 signaling pathway.

This study, using a network pharmacologic approach, provides another strategy that can help us to understand the mechanisms by which TW treats AML comprehensively.

## 1 Introduction

Acute myeloid leukemia (AML) is one of the common tumors in the blood system and is caused by the cloning and amplification of undifferentiated myeloid progenitor cells.^[1,2]^ Impaired hematopoietic function and bone marrow failure are the most important features of AML, which can lead to death.^[3]^ Although advances in AML treatment have significantly improved the prognosis of young patients, a mass of the new cases are elderly, and the prognosis of the elderly is still very poor.^[4]^ Even with current treatments, the prognosis is poor, with a 5-year relative survival rate of only 30% to 50%.^[5]^

Traditional Chinese medicine (TCM) doctors in China have been using TCM clinically for more than 2500 years.^[6]^ Some TCMs have been proven to have therapeutic effects on AML, such as arsenic trioxide.^[7]^ And some other experiments also proved that TCM has a therapeutic effect on AML.^[8]^*Tripterygium wilfordii* (TW), as one of the TCMs, there is evidence to show that the TCMs have anti-tumor ability.^[9]^ Proved by research, through mitochondrial-mediated pathway, triptolide can cause apoptosis of AML cells.^[10]^ Although the active ingredients in TW have been identified, its relevant mechanism of anti-AML pharmacological effects remains to be studied. In recent years, not only the continuous development of system biology, but also network pharmacology and computer technology are booming. Network pharmacology is a new discipline based on the theory of systems biology, which analyzes the network of biological systems and selects specific signal nodes (Nodes) for multitarget drug molecular design. Network pharmacology emphasizes the multichannel regulation of signal pathways, improves the therapeutic effect of drugs, reduces toxic side effects, thereby increasing the success rate of clinical trials of new drugs and saving drug development costs.^[11]^ Therefore as a useful tool, it could assist us to further understand what the drugs can do, and help us to deepen our insights on how to improve the discovery of drugs in diseases.^[12,13]^

**Figure 1:**
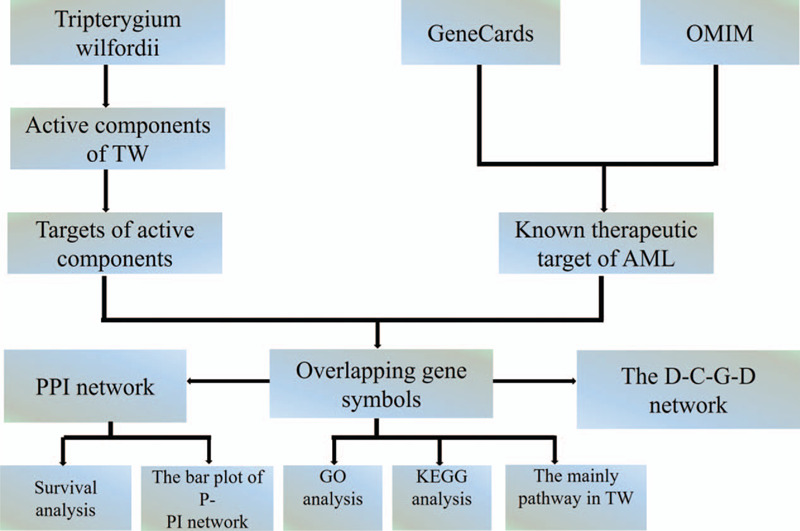
Flow chart of this study. D-C-G-D = Drug-Compounds-Genes-Disease; GO = Gene Ontology; OMIM = Online Mendelian Inheritance in Man; PPI = protein-protein interaction, TW = *Tripterygium wilfordii*.

In this study, the potential mechanism of TW treatment AML was analyzed by the method of network pharmacology. The target protein of TW active ingredient and the target of AML treatment were intersected. With TW and AML as the center, Drug-Compounds-Genes-Disease Network was constructed. Construct a protein-protein interaction (PPI) network based on overlapping genes, and conduct enrichment analysis of GO and KEGG pathways. We hope to explore TW's potential therapeutic targets in anti-AML, and provide a basis for the study of TW pharmacological mechanism (Fig. 1).

## 2 Method

### 2.1 Screening of active compounds and target proteins

The active compounds of TW and their target proteins were obtained from the Traditional Chinese Medicine Systems Pharmacology Database and Analysis Platform (TCMSP, http://tcmspw.com). As a special database that contains a lot of herbs, active constituents, and their targets, TCMSP is established by using the pharmacology of TCM systems.^[14]^ It also includes some pharmacokinetic properties of active compounds, such as oral bioavailability (OB), drug-likeness (DL), and others. OB, as one of the most important pharmacokinetic parameters, indicates the ratio of the drug taken to the blood circulation.^[15]^ The larger the OB value of compounds, the more likely it is to become a clinical drug. The similarity between a compound and a drug which is already known can be expressed in terms of DL.^[16]^ It represents that although the compounds is not a drug, it has the possibility of becoming a drug. Therefore, the greater the value of DL, the greater the possibility that this ingredient will become a potential therapeutic drug for AML. The larger the value of OB, the higher the effectiveness of the ingredient in treating AML.

So, among these pharmacokinetic properties, OB and DL are considered to be the most important evaluation indicators of whether they can become drugs.^[17,18]^ Due to the poor pharmacological activity of most compounds in TCM, they cannot effectively act on the target protein. Therefore, OB ≥30% and DL ≥0.18, which are considered as indicators for screening a clinical drug.^[19,20]^ In our study, we also used those indicators as our screening criteria.

### 2.2 The acquisition of gene targets for acute myeloid leukemia

The target genes for AML in this study were collected from 2 databases. The first database is Gene Cards (https://www.genecards.org/, version 4.9.0), as a database that contains a lot of genes, such as genome, transcriptome, proteome and genetics, as well as clinical and functional information from 150 web sources.^[21]^ The other database is the Online Mendelian Inheritance in Man (OMIM) database (http://www.omim.org/).^[22]^

### 2.3 Drug-compounds-genes-disease (D-C-G-D) network construction

At the beginning, we took the target genes of TW's active component and the therapeutic targets for AML obtained from the above 2 databases to get the overlapping genes. Subsequently, we integrated the information of the component, drug, genetic, diseases. Finally, we used Cytoscape software (v.3.6.1, https://cytoscape.org/) to visually analyze these data to construct D-C-G-D.^[23,24]^

### 2.4 PPI networks construction

PPI data were attained from the STRING database (https://string-db.org/, version 11.0), which is a database that can predict known protein interactions.^[25]^

### 2.5 Survival analysis

The information for these gene survival analyses comes from the OncoLnc database (http://www.oncolnc.org/). It contains RNA-SEQ expression and survival data of 8647 patients’ mRNAs and miRNAs in 21 cancer studies, which are conducted by the Cancer Genome Atlas (TCGA).^[26]^

### 2.6 Gene ontology enrichment analysis

In this study, processed by DAVID6.8 (https://david.ncifcrf.gov/), every genes’ GO as well as KEGG enrichment analysis were obtained. Three aspects are contained in GO enrichment analysis: biological process (BP), molecular function (MF), and cellular component (CC).^[27,28]^ All the GO and KEGG enrichment analysis results were selected by *P* value less than .05 as the critical criterion.

## 3 Results

### 3.1 Screening of active compounds and targets

To get the active compounds of TW, OB and DL, which are 2 ADME parameters, were used for screening the data that were downloaded from TCMSP. By using the value of OB that is greater than 30 and the value of DL that is greater than 0.18 as the standard, we obtained 53 active compounds (Supplementary 1). Then, we downloaded the data about 53 active compounds’ target. After deleting the useless items, 64 known targets were finally obtained. The details of these target are shown in Supplementary 2.

### 3.2 The collection of known therapeutic targets for AML

From Gene Card database, total 11,619 Therapeutic targets, which are already known for AML, were acquired. In order to improve the accuracy of our data, Gifts greater than 10 were used as a standard to streamline the data. Then, 1093 Known therapeutic targets were collected. Finally, we downloaded 348 known therapeutic targets from OMIM to enrich our data.

### 3.3 Screening the overlapping gene and constructing D-C-G-D network

**Figure 2:**
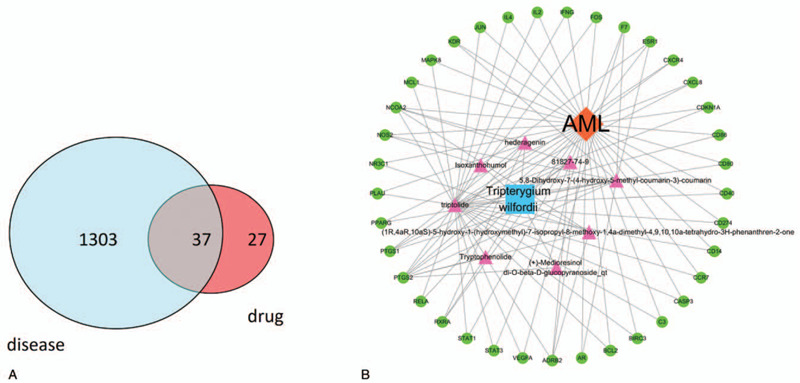
(A) The 37 overlapping genes between the disease and drug. (B) The Drug-Compounds-Genes-Disease network.

By taking the intersection of drug targets and disease targets, we got 37 genes (Fig. 2A) and 8 active compounds (Table 1), which means that these genes could play a major role in TW treatment of AML. In order to clarify the potential mechanism of TW's effect on AML, we constructed a D-C-G-D network by Cytoscape software (Fig. 2B).

**Table 1:** Eight active compounds from *Tripterygium wilfordii* (TW) and their corresponding predicted oral bioavailability (OB), drug-likeliness (DL).

Mol ID	Molecule name	OB (%)	DL
MOL000296	hederagenin	36.91	0.75
MOL003182	(+)-Medioresinol di-O-beta-D-glucopyranoside_qt	60.69	0.62
MOL003184	81827-74-9	45.42	0.53
MOL003185	(1R,4aR,10aS)-5-hydroxy-1-(hydroxymethyl)-7-isopropyl-8-methoxy-1,4a-dimethyl-4,9,10,10a-tetrahydro-3H-phenanthren-2-one	48.84	0.38
MOL003187	triptolide	51.29	0.68
MOL003196	Tryptophenolide	48.5	0.44
MOL003199	5,8-Dihydroxy-7-(4-hydroxy-5-methyl-coumarin-3)-coumarin	61.85	0.54
MOL003217	Isoxanthohumol	56.81	0.39

### 3.4 Construction and analysis of target proteins PPI network

**Figure 3:**
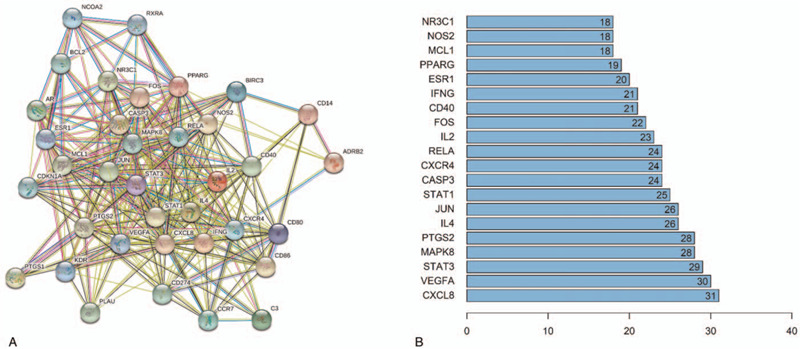
(A) The protein-protein interaction (PPI) network of 37 overlapping genes. (B) The bar plot of the top 20 genes in the PPI network.

For the purpose of figuring out how overlapping genes interact, we uploaded the information of them to the STRING database and then structure a PPI network, which contained 37 nodes and 324 edges (Fig. 3A). And we show the bar plot with the top 20 genes in the PPI network in Figure 3B. The result of PPI network indicates that there is a complex relationship between these genes.

### 3.5 Survival analysis

**Figure 4:**
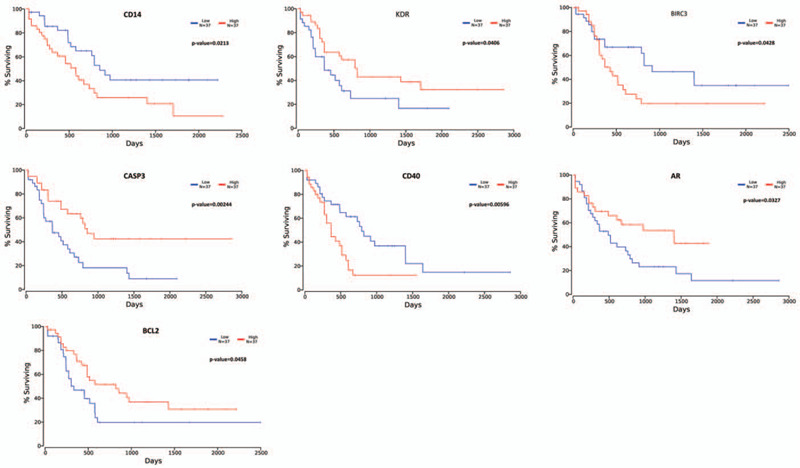
(A) Survival analysis of part of these target proteins.

To clarify the impact of these genes on survival and prognosis of AML, survival analysis was performed. The results of *P* < .05 are shown in Figure 4; the results indicated that some genes play an important role in the AML patients’ survival and prognosis. The result suggested that some active compounds in TW could act on these genes to improve the prognosis of patients.

### 3.6 Gene ontology enrichment analysis

**Figure 5:**
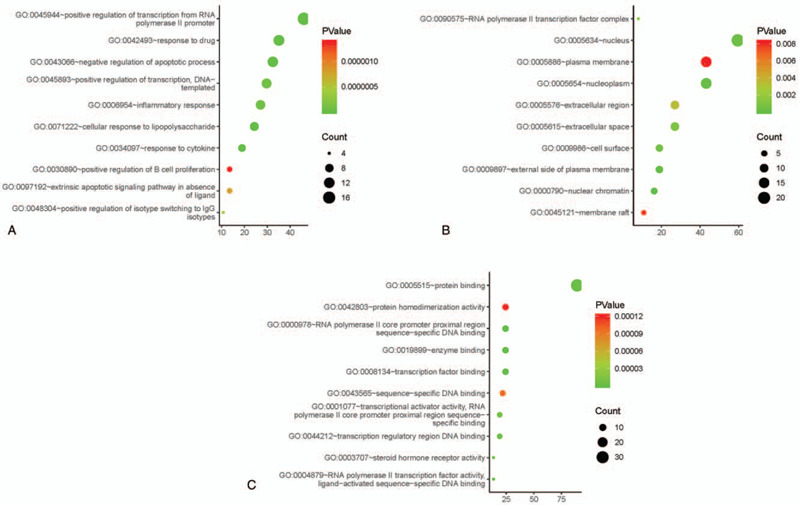
Gene Ontology enrichment analysis of 37 overlapping genes: (A) top 10 significantly enriched biological process; (B) top 10 significantly enriched cellular component; (C) top 10 significantly enriched molecular function.

For the purpose of figuring out what the relationships between 37 genes and AML are, we carried out GO enrichment analysis on these genes in DAVID database and the result of GO enrichment analysis included 3 different levels: BP, MF, and CC. We selected the top 10 according to the *P* value from small to large as shown in Figure 5. The results indicated that TW treats AML through various biological processes, including reaction to drug (GO:0042493), negative regulation of apoptotic process (GO:0043066), reaction to cytokine (GO:0034097), and others. In the CC classification, nucleus is the main classification of the target proteins. The enrichment analysis results indicated that the active ingredients of TW have ability to affect the transcription of AML cell genes to treat AML.

### 3.7 KEGG enrichment analysis

**Figure 6:**
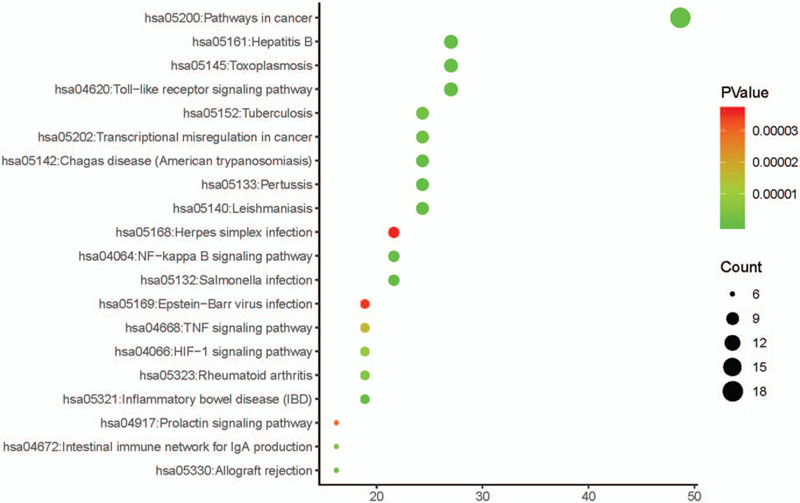
KEGG pathway enrichment analysis of 37 overlapping genes the top 20 significantly enriched pathways.

To further explore how TW affects AML through these target genes, we uploaded these 37 genes to the DAVID database and performed KEGG enrichment analysis. And we selected the top 10 according to the *P* value from small to large as shown in Figure 6. KEGG pathway analysis of these genes presented several pathways correlative with the development and treatment of AML, including Pathways in cancer (hsa05200), NF-kappa B signaling pathway (hsa04064), Transcriptional misregulation in cancer (hsa05202), HIF-1 signaling pathway (hsa04066), and others. We can find that a pathway includes multiple proteins, and a protein is also involved in multiple pathways. This complex regulatory relationship indicates that the active pharmacological components in TW may be used to treat some diseases including AML through these signaling pathways.

## 4 Discussion

It is well-known that Chinese medicine has been utilized in various illnesses treatment for long times in China. In the research of active pharmaceutical ingredients, the method based on network interactions is of great help in deepening the understanding of the role of drugs in multilayer information.^[29]^ The interaction network of drugs, active ingredients, diseases, and target proteins is an important way to express the mechanism of drugs and their active ingredients in the treatment of a certain disease, which helps to deepen the understanding of the therapeutic effects of drugs and offer a theoretical basis for new drugs development.^[30]^ As an important pharmacological research method, network pharmacology can better demonstrate the relationship between drugs, components, and targets. The application of this idea in the research of TCM can help to understand the interaction mechanism of multicomponent multitarget model of TCM.^[31]^

In this research, by taking the intersection of the active ingredient target of TW and the target of AML treatment, we obtained 37 target genes and 8 active compounds. In these 8 active compounds, Triptolide has been shown to have a therapeutic effect on AML.^[9,10]^ Triptolide can inhibit the expression of MDM2 and lead to the p53-dependent pathway inhibition and thus achieve the curative effect on AML.^[32]^ Other active ingredients in TW have also been shown to have therapeutic effects on other cancers. Such as Hederagenin, it can inhibit the proliferation of cervical cancer CaSki cells and promote their apoptosis by blocking the STAT3 pathway.^[33]^ And Isoxanthohumol can inhibit the formation of lung metastasis in B16-F10 murine melanoma model.^[34]^ Tripterygium also exhibited significant inhibitory effects on many cancer cells.^[35]^

On the contrary, it can be seen from the D-C-G-D Network that these active ingredients have a common target, which shows that they have some synergistic effects in the treatment of AML. As one of the predicted targets of TW for the treatment of AML, PTGS2 can be acted by 7 active compounds in TW. It has been reported that it can selectively inhibit cox-2 and play a role in the treatment of AML.^[35–37]^ This means that multiple active ingredients in TCM can act on a single target to take effect in the treatment of diseases. As for the active component, Triptolide is associated with 25 target genes, such as PTGS2, CD40, CASP3, JUN, etc, which is consistent with the fact that a single active compound can act on multiple targets.

In order to clarify the impact of these genes on the survival and prognosis of patients, we conducted a survival analysis of these 37 genes. The results show that some of these genes have a significant impact on the survival and prognosis of patients. And some genes have been experimentally proven to be the target of TW treatment of diseases such as AR, CD14, BCL2.^[38–40]^ This means that these genes may be the crucial target of TW in the treatment of leukemia.

Next, we conducted GO and KEGG enrichment analysis on these target genes. The major signaling pathways related to AML were found to include Pathways in cancer, Toll-like receptor signaling pathway, NF-kappa B signaling pathway, Transcriptional misregulation in cancer, HIF-1 signaling pathway, and other pathways closely connected to the occurrence and development of AML. This indicates that TW may have multiple active ingredients through multiple pathways to prevent and treat AML. Such as Toll-like receptor signaling pathway, as a part of the innate immune system, studies have shown that various hematological malignant cells, including AML cells, express TLRs, and TLR agonists may directly effect on the leukemic cells.^[37]^ Due to the indirect anti-leukemia effect caused by the activation/stimulation of normal immuneocompetent cells, such as eradicating residual leukemia cells after induction therapy, TLR is considered a possible drug target in AML.^[41]^ In addition, the release of pro-inflammatory cytokines due to TLR stimulation can enhance the immunogenicity of AML blasts, making it easier to treat.^[42]^ It is well known that NF-κB is constitutively activated in most patients with AML, leading to apoptosis resistant. Studies have shown that inhibiting NF-κB can induce apoptosis in AML cells.^[43]^ Studies have shown that HIF1α promotes the proliferation of AML cells through a pro-tumoral chemokine factor signaling pathway.^[44]^

Due to the complexity of TCM ingredients and the limitations of experimental research methods, there are no reports on the effective ingredients, targets, and mechanisms of TW for AML treatment. This study shows that using network pharmacology to study the effective ingredients and therapeutic targets of TW in the treatment of AML is of great significance for deepening the understanding of its related mechanism of action and discovering new effective ingredients. For the first time, the multitargets and multipathways that play a role in AML are used to explain the molecular mechanism of TW's therapeutic effect. However, the pathogenesis of AML is more complicated. Signaling pathways cross and regulate each other, presenting a pattern of multiple pathways and multiple targets. There is a dose--effect relationship between drugs and diseases, and the current network pharmacology technology is difficult to achieve the purpose of quantification. Finally, most of the research based on network pharmacology is still in static network analysis, and body function is a continuous dynamic process; the occurrence of diseases, drug development, and efficacy process are also dynamic changes.^[45]^ In-depth analysis of the anti-AML pharmacological actions of TW, as well as the targets and pathways of the active ingredients are necessary to be further verified. And a large number of experimental verifications in vivo or in vitro are required.

## 5 Conclusion

In this study, 8 active compounds with TW between AML and 37 overlapping genes were selected by the method of network pharmacology. After that, we constructed a PPI network of these genes, and conducted a survival analysis on the top 20 nodes to identify the effect of these genes on patients’ survival and prognosis. The enrichment analysis of GO and KEGG was conducted to find the potential mechanism of action of TW in AML treatment. In conclusion, this study provides another strategy to understand the mechanisms comprehensively by which TW treats AML.

## Author contributions

Wenjun Liu and Tingting Fang conceived and designed the studies. Lanqin Liu participated in this work. All authors participated in drafting of the manuscript and revising it before final submission.

**Conceptualization:** Wenjun Liu.

**Funding acquisition:** Wenjun Liu.

**Investigation:** Tingting Fang, Lanqin Liu, Wenjun Liu.

**Methodology:** Tingting Fang.

**Resources:** Tingting Fang, Lanqin Liu.

**Software:** Tingting Fang, Lanqin Liu.

**Supervision:** Wenjun Liu.

**Validation:** Tingting Fang, Lanqin Liu, Wenjun Liu.

**Writing – original draft:** Tingting Fang.

**Writing – review & editing:** Wenjun Liu.

## Supplementary Material

Supplemental Digital Content

## Supplementary Material

Supplemental Digital Content
