# Supplementary material for: Network pharmacology-based strategy for predicting therapy targets of Tripterygium wilfordii on acute myeloid leukemia
Source: Medicine (Baltimore). 2020 Dec 11;99(50):e23546. doi: 10.1097/MD.0000000000023546 (PMC7738111; doi:10.1097/MD.0000000000023546)
Supplement: Supplemental Digital Content [file medi-99-e23546-s001.pdf]

Supplementary Table S1. Ingredients information of Tripterygium wilfordii.

Mol ID	Molecule Name	OB (%)	DL
MOL000296	hederagenin	36.91	0.75
MOL003182	(+)-Medioresinol di-O-beta-D-glucopyranoside_qt	60.69	0.62
MOL003184	81827-74-9	45.42	0.53
MOL003185	(1R,4aR,10aS)-5-hydroxy-1-(hydroxymethyl)-7-isopropyl-8-methoxy-1,4a-dimethyl-4,9,10,10a-tetrahydro-3H-phenanthren-2-one	48.84	0.38
MOL003187	triptolide	51.29	0.68
MOL003188	Tripchlorolide	78.72	0.72
MOL003189	WILFORLIDE A	35.66	0.72
MOL003192	Triptonide	67.66	0.7
MOL003196	Tryptophenolide	48.5	0.44
MOL003198	5 alpha-Benzoyl-4 alpha-hydroxy-1 beta,8 alpha-dinicotinoyl-dihydro-agarofuran	35.26	0.72
MOL003199	5,8-Dihydroxy-7-(4-hydroxy-5-methyl-coumarin-3)-coumarin	61.85	0.54
MOL003206	Canin	77.41	0.33
MOL003208	Celafurine	72.94	0.44
MOL003209	Celallocinnine	83.47	0.59
MOL003210	Celapanine	30.18	0.82
MOL003211	Celaxanthin	47.37	0.58
MOL003217	Isoxanthohumol	56.81	0.39
MOL003222	Salazinic acid	36.34	0.76
MOL003224	Tripdiotolnide	56.4	0.67
MOL003225	Hypodiolide A	76.13	0.49

MOL003229	Triptinin B	34.73	0.32
MOL003231	Triptoditerpenic acid B	40.02	0.36
MOL003232	Triptofordin B1	39.55	0.84
MOL003233	Triptofordin B2	107.71	0.76
MOL003234	Triptofordin C2	30.16	0.76
MOL003235	Triptofordin D1	32	0.75
MOL003236	Triptofordin D2	30.38	0.69
MOL003238	Triptofordin F1	33.91	0.6
MOL003239	Triptofordin F2	33.62	0.67
MOL003241	Triptofordin F4	31.37	0.67
MOL003242	Triptofordinine A2	30.78	0.47
MOL003244	Triptonide	68.45	0.68
MOL003245	Triptonoditerpenic acid	42.56	0.39
MOL003248	Triptonoterpene	48.57	0.28
MOL003266	21-Hydroxy-30-norhopan-22-one	34.11	0.77
MOL003267	Wilformine	46.32	0.2
MOL003278	salaspermic acid	32.19	0.63
MOL003279	99694-86-7	75.23	0.66
MOL003280	TRIPTONOLIDE	49.51	0.49
MOL000358	beta-sitosterol	36.91	0.75
MOL000211	Mairin	55.38	0.78
MOL000422	kaempferol	41.88	0.24
MOL000449	Stigmasterol	43.83	0.76

MOL002058	40957-99-1	57.2	0.62
MOL003283	(2R,3R,4S)-4-(4-hydroxy-3-methoxy-phenyl)-7-methoxy-2,3-dimethylol-tetralin-6-ol	66.51	0.39
MOL004443	Zhebeiresinol	58.72	0.19
MOL005828	nobiletin	61.67	0.52
MOL007415	[(2S)-2-[[(2S)-2-(benzoylamino)-3-phenylpropanoyl]amino]-3-phenylpropyl] acetate	58.02	0.52
MOL007535	(5S,8S,9S,10R,13R,14S,17R)-17-[(1R,4R)-4-ethyl-1,5-dimethylhexyl]-10,13-dimethyl-2,4,5,7,8,9,11,12,14,15,16,17-dodecahydro-1H-cyclopenta[a]phenanthrene-3,6-dione	33.12	0.79
MOL009386	3,3'-bis-(3,4-dihydro-4-hydroxy-6-methoxy)-2H-1-benzopyran	52.11	0.54
MOL011169	Peroxyergosterol	44.39	0.82
