# Supplementary material for: Network pharmacology-based strategy for predicting therapy targets of Tripterygium wilfordii on acute myeloid leukemia
Source: Medicine (Baltimore). 2020 Dec 11;99(50):e23546. doi: 10.1097/MD.0000000000023546 (PMC7738111; doi:10.1097/MD.0000000000023546)
Supplement: Supplemental Digital Content [file medi-99-e23546-s002.pdf]

Supplementary Table S2. target genes of active compounds

MolId	MolName	Target
MOL000296	hederagenin	Progesterone receptor
MOL000296	hederagenin	Nuclear receptor coactivator 2
MOL000296	hederagenin	Muscarinic acetylcholine receptor M3
MOL000296	hederagenin	Muscarinic acetylcholine receptor M1
MOL000296	hederagenin	Gamma-aminobutyric-acid receptor alpha-2 subunit
MOL000296	hederagenin	Gamma-aminobutyric-acid receptor alpha-3 subunit
MOL000296	hederagenin	Muscarinic acetylcholine receptor M2
MOL000296	hederagenin	Alpha-1B adrenergic receptor
MOL000296	hederagenin	Gamma-aminobutyric acid receptor subunit alpha-1
MOL000296	hederagenin	Glutamate receptor 2
MOL000296	hederagenin	Gamma-aminobutyric-acid receptor subunit alpha-6
MOL000296	hederagenin	Gamma-aminobutyric-acid receptor alpha-5 subunit
MOL000296	hederagenin	Ig gamma-1 chain C region
MOL000296	hederagenin	Alcohol dehydrogenase 1B
MOL000296	hederagenin	Alcohol dehydrogenase 1C
MOL000296	hederagenin	Lysozyme
MOL000296	hederagenin	Nicotinate-nucleotide--dimethylbenzimidazole phosphoribosyltransferase
MOL000296	hederagenin	Prostaglandin G/H synthase 1
MOL000296	hederagenin	Sodium channel protein type 5 subunit alpha
MOL000296	hederagenin	Prostaglandin G/H synthase 2
MOL000296	hederagenin	Retinoic acid receptor RXR-alpha
MOL000296	hederagenin	CGMP-inhibited 3',5'-cyclic phosphodiesterase A
MOL000296	hederagenin	Sodium-dependent noradrenaline transporter
MOL000296	hederagenin	Cytochrome P450-cam
MOL003182	(+)-Medioresinol di-O-beta-D-glucopyranoside_qt	Potassium voltage-gated channel subfamily H member 2
MOL003182	(+)-Medioresinol di-O-beta-D-glucopyranoside_qt	Sodium channel protein type 5 subunit alpha
MOL003182	(+)-Medioresinol di-O-beta-D-glucopyranoside_qt	Coagulation factor Xa
MOL003182	(+)-Medioresinol di-O-beta-D-glucopyranoside_qt	Prostaglandin G/H synthase 2
MOL003182	(+)-Medioresinol di-O-beta-D-glucopyranoside_qt	Nitric-oxide synthase, endothelial
MOL003182	(+)-Medioresinol di-O-beta-D-glucopyranoside_qt	Coagulation factor VII

MOL003182	(+)-Medioresinol di-O-beta-D-glucopyranoside_qt	DNA topoisomerase II
MOL003182	(+)-Medioresinol di-O-beta-D-glucopyranoside_qt	Heat shock protein HSP 90
MOL003182	(+)-Medioresinol di-O-beta-D-glucopyranoside_qt	Calmodulin
MOL003184	81827-74-9	Prostaglandin G/H synthase 1
MOL003184	81827-74-9	Muscarinic acetylcholine receptor M3
MOL003184	81827-74-9	Potassium voltage-gated channel subfamily H member 2
MOL003184	81827-74-9	Muscarinic acetylcholine receptor M1
MOL003184	81827-74-9	Sodium channel protein type 5 subunit alpha
MOL003184	81827-74-9	Coagulation factor Xa
MOL003184	81827-74-9	Muscarinic acetylcholine receptor M5
MOL003184	81827-74-9	Prostaglandin G/H synthase 2
MOL003184	81827-74-9	Carbonic anhydrase II
MOL003184	81827-74-9	Muscarinic acetylcholine receptor M4
MOL003184	81827-74-9	Delta-type opioid receptor
MOL003184	81827-74-9	Progesterone receptor
MOL003184	81827-74-9	Muscarinic acetylcholine receptor M2
MOL003184	81827-74-9	Alpha-1B adrenergic receptor
MOL003184	81827-74-9	Beta-2 adrenergic receptor
MOL003184	81827-74-9	DNA topoisomerase II
MOL003184	81827-74-9	Mu-type opioid receptor
MOL003184	81827-74-9	Heat shock protein HSP 90
MOL003184	81827-74-9	Nuclear receptor coactivator 2
MOL003184	81827-74-9	Nuclear receptor coactivator 1
MOL003184	81827-74-9	Calcium-activated potassium channel subunit alpha 1
MOL003185	(1R,4aR,10aS)-5-hydroxy-1-(hydroxymethyl)-7-isopropyl-8-methoxy-1,4a-dimethyl-4,9,10,10a-tetrahydro-3H-phenanthren-2-one	Muscarinic acetylcholine receptor M3
MOL003185	(1R,4aR,10aS)-5-hydroxy-1-(hydroxymethyl)-7-isopropyl-8-methoxy-1,4a-dimethyl-4,9,10,10a-tetrahydro-3H-phenanthren-2-one	Muscarinic acetylcholine receptor M1
MOL003185	(1R,4aR,10aS)-5-hydroxy-1-(hydroxymethyl)-7-isopropyl-8-methoxy-1,4a-dimethyl-4,9,10,10a-tetrahydro-3H-phenanthren-2-one	Prostaglandin G/H synthase 2

MOL003185	(1R,4aR,10aS)-5-hydroxy-1-(hydroxymethyl)-7-isopropyl-8-methoxy-1,4a-dimethyl-4,9,10,10a-tetrahydro-3H-phenanthren-2-one	Carbonic anhydrase II
MOL003185	(1R,4aR,10aS)-5-hydroxy-1-(hydroxymethyl)-7-isopropyl-8-methoxy-1,4a-dimethyl-4,9,10,10a-tetrahydro-3H-phenanthren-2-one	Delta-type opioid receptor
MOL003185	(1R,4aR,10aS)-5-hydroxy-1-(hydroxymethyl)-7-isopropyl-8-methoxy-1,4a-dimethyl-4,9,10,10a-tetrahydro-3H-phenanthren-2-one	Alpha-1A adrenergic receptor
MOL003185	(1R,4aR,10aS)-5-hydroxy-1-(hydroxymethyl)-7-isopropyl-8-methoxy-1,4a-dimethyl-4,9,10,10a-tetrahydro-3H-phenanthren-2-one	Alpha-1B adrenergic receptor
MOL003185	(1R,4aR,10aS)-5-hydroxy-1-(hydroxymethyl)-7-isopropyl-8-methoxy-1,4a-dimethyl-4,9,10,10a-tetrahydro-3H-phenanthren-2-one	Alpha-1D adrenergic receptor
MOL003185	(1R,4aR,10aS)-5-hydroxy-1-(hydroxymethyl)-7-isopropyl-8-methoxy-1,4a-dimethyl-4,9,10,10a-tetrahydro-3H-phenanthren-2-one	DNA topoisomerase II
MOL003185	(1R,4aR,10aS)-5-hydroxy-1-(hydroxymethyl)-7-isopropyl-8-methoxy-1,4a-dimethyl-4,9,10,10a-tetrahydro-3H-phenanthren-2-one	Mu-type opioid receptor

MOL003185	(1R,4aR,10aS)-5-hydroxy-1-(hydroxymethyl)-7-isopropyl-8-methoxy-1,4a-dimethyl-4,9,10,10a-tetrahydro-3H-phenanthren-2-one	Glucocorticoid receptor
MOL003185	(1R,4aR,10aS)-5-hydroxy-1-(hydroxymethyl)-7-isopropyl-8-methoxy-1,4a-dimethyl-4,9,10,10a-tetrahydro-3H-phenanthren-2-one	Nuclear receptor coactivator 2
MOL003185	(1R,4aR,10aS)-5-hydroxy-1-(hydroxymethyl)-7-isopropyl-8-methoxy-1,4a-dimethyl-4,9,10,10a-tetrahydro-3H-phenanthren-2-one	Nuclear receptor coactivator 1
MOL003185	(1R,4aR,10aS)-5-hydroxy-1-(hydroxymethyl)-7-isopropyl-8-methoxy-1,4a-dimethyl-4,9,10,10a-tetrahydro-3H-phenanthren-2-one	Sodium channel protein type 5 subunit alpha
MOL003185	(1R,4aR,10aS)-5-hydroxy-1-(hydroxymethyl)-7-isopropyl-8-methoxy-1,4a-dimethyl-4,9,10,10a-tetrahydro-3H-phenanthren-2-one	Muscarinic acetylcholine receptor M2
MOL003185	(1R,4aR,10aS)-5-hydroxy-1-(hydroxymethyl)-7-isopropyl-8-methoxy-1,4a-dimethyl-4,9,10,10a-tetrahydro-3H-phenanthren-2-one	Beta-2 adrenergic receptor
MOL003185	(1R,4aR,10aS)-5-hydroxy-1-(hydroxymethyl)-7-isopropyl-8-methoxy-1,4a-dimethyl-4,9,10,10a-tetrahydro-3H-phenanthren-2-one	Heat shock protein HSP 90
MOL003187	triptolide	Transcription factor p65
MOL003187	triptolide	Signal transducer and activator of transcription 3
MOL003187	triptolide	Vascular endothelial growth factor A
MOL003187	triptolide	Apoptosis regulator Bcl-2

MOL003187	triptolide	Proto-oncogene c-Fos
MOL003187	triptolide	Cyclin-dependent kinase inhibitor 1
MOL003187	triptolide	Urokinase-type plasminogen activator
MOL003187	triptolide	Tumor necrosis factor
MOL003187	triptolide	Transcription factor AP-1
MOL003187	triptolide	Caspase-3
MOL003187	triptolide	Cellular tumor antigen p53
MOL003187	triptolide	Mitogen-activated protein kinase 8
MOL003187	triptolide	Prostaglandin G/H synthase 2
MOL003187	triptolide	Signal transducer and activator of transcription 1-alpha/beta
MOL003187	triptolide	Interleukin-8
MOL003187	triptolide	Induced myeloid leukemia cell differentiation protein Mcl-1
MOL003187	triptolide	Transforming growth factor beta-1
MOL003187	triptolide	Interleukin-2
MOL003187	triptolide	Interferon gamma
MOL003187	triptolide	Interleukin-4
MOL003187	triptolide	Baculoviral IAP repeat-containing protein 4
MOL003187	triptolide	Beta-defensin 2
MOL003187	triptolide	T-lymphocyte activation antigen CD80
MOL003187	triptolide	T-lymphocyte activation antigen CD86
MOL003187	triptolide	C-X-C chemokine receptor type 4
MOL003187	triptolide	Baculoviral IAP repeat-containing protein 3
MOL003187	triptolide	Programmed cell death 1 ligand 1
MOL003187	triptolide	Interleukin-23 subunit alpha
MOL003187	triptolide	C-C chemokine receptor type 7
MOL003187	triptolide	T-cell surface glycoprotein CD1a
MOL003187	triptolide	Tumor necrosis factor receptor superfamily member 5
MOL003187	triptolide	Monocyte differentiation antigen CD14
MOL003187	triptolide	Complement C3
MOL003187	triptolide	V-set domain-containing T-cell activation inhibitor 1
MOL003196	Tryptophenolide	Dopamine D1 receptor
MOL003196	Tryptophenolide	Muscarinic acetylcholine receptor M3
MOL003196	Tryptophenolide	Potassium voltage-gated channel subfamily H member 2
MOL003196	Tryptophenolide	Muscarinic acetylcholine receptor M1
MOL003196	Tryptophenolide	Sodium channel protein type 5 subunit alpha
MOL003196	Tryptophenolide	Muscarinic acetylcholine receptor M5
MOL003196	Tryptophenolide	Prostaglandin G/H synthase 2
MOL003196	Tryptophenolide	Carbonic anhydrase II
MOL003196	Tryptophenolide	Retinoic acid receptor RXR-alpha
MOL003196	Tryptophenolide	Delta-type opioid receptor
MOL003196	Tryptophenolide	CGMP-inhibited 3',5'-cyclic phosphodiesterase A
MOL003196	Tryptophenolide	Alpha-1A adrenergic receptor
MOL003196	Tryptophenolide	Progesterone receptor
MOL003196	Tryptophenolide	Muscarinic acetylcholine receptor M2
MOL003196	Tryptophenolide	Alpha-1B adrenergic receptor

MOL003196	Tryptophenolide	Beta-2 adrenergic receptor
MOL003196	Tryptophenolide	Alpha-1D adrenergic receptor
MOL003196	Tryptophenolide	DNA topoisomerase II
MOL003196	Tryptophenolide	Mu-type opioid receptor
MOL003196	Tryptophenolide	Heat shock protein HSP 90
MOL003196	Tryptophenolide	Neuronal acetylcholine receptor protein, alpha-7 chain
MOL003196	Tryptophenolide	Nuclear receptor coactivator 2
MOL003196	Tryptophenolide	Nuclear receptor coactivator 1
MOL003199	5,8-Dihydroxy-7-(4-hydroxy-5-methyl-coumarin-3)-coumarin	Nitric oxide synthase, inducible
MOL003199	5,8-Dihydroxy-7-(4-hydroxy-5-methyl-coumarin-3)-coumarin	Prostaglandin G/H synthase 1
MOL003199	5,8-Dihydroxy-7-(4-hydroxy-5-methyl-coumarin-3)-coumarin	Thrombin
MOL003199	5,8-Dihydroxy-7-(4-hydroxy-5-methyl-coumarin-3)-coumarin	Potassium voltage-gated channel subfamily H member 2
MOL003199	5,8-Dihydroxy-7-(4-hydroxy-5-methyl-coumarin-3)-coumarin	Estrogen receptor
MOL003199	5,8-Dihydroxy-7-(4-hydroxy-5-methyl-coumarin-3)-coumarin	Androgen receptor
MOL003199	5,8-Dihydroxy-7-(4-hydroxy-5-methyl-coumarin-3)-coumarin	Sodium channel protein type 5 subunit alpha
MOL003199	5,8-Dihydroxy-7-(4-hydroxy-5-methyl-coumarin-3)-coumarin	Peroxisome proliferator activated receptor gamma
MOL003199	5,8-Dihydroxy-7-(4-hydroxy-5-methyl-coumarin-3)-coumarin	Coagulation factor Xa
MOL003199	5,8-Dihydroxy-7-(4-hydroxy-5-methyl-coumarin-3)-coumarin	Prostaglandin G/H synthase 2
MOL003199	5,8-Dihydroxy-7-(4-hydroxy-5-methyl-coumarin-3)-coumarin	Coagulation factor VII
MOL003199	5,8-Dihydroxy-7-(4-hydroxy-5-methyl-coumarin-3)-coumarin	Vascular endothelial growth factor receptor 2
MOL003199	5,8-Dihydroxy-7-(4-hydroxy-5-methyl-coumarin-3)-coumarin	Dipeptidyl peptidase IV
MOL003199	5,8-Dihydroxy-7-(4-hydroxy-5-methyl-coumarin-3)-coumarin	Glycogen phosphorylase, muscle form
MOL003199	5,8-Dihydroxy-7-(4-hydroxy-5-methyl-coumarin-3)-coumarin	Heat shock protein HSP 90

MOL003199	5,8-Dihydroxy-7-(4-hydroxy-5-methyl-coumarin-3)-coumarin	Cell division protein kinase 2
MOL003199	5,8-Dihydroxy-7-(4-hydroxy-5-methyl-coumarin-3)-coumarin	Trypsin-1
MOL003199	5,8-Dihydroxy-7-(4-hydroxy-5-methyl-coumarin-3)-coumarin	Calmodulin
MOL003208	Celafurine	Thrombin
MOL003209	Celallocinnine	Potassium voltage-gated channel subfamily H member 2
MOL003209	Celallocinnine	Sodium channel protein type 5 subunit alpha
MOL003209	Celallocinnine	Coagulation factor Xa
MOL003217	Isoxanthohumol	Nitric oxide synthase, inducible
MOL003217	Isoxanthohumol	Potassium voltage-gated channel subfamily H member 2
MOL003217	Isoxanthohumol	Estrogen receptor
MOL003217	Isoxanthohumol	Sodium channel protein type 5 subunit alpha
MOL003217	Isoxanthohumol	Coagulation factor Xa
